# Changes in Diffusion Kurtosis Imaging and Magnetic Resonance Spectroscopy in a Direct Cranial Blast Traumatic Brain Injury (dc-bTBI) Model

**DOI:** 10.1371/journal.pone.0136151

**Published:** 2015-08-24

**Authors:** Jiachen Zhuo, Kaspar Keledjian, Su Xu, Adam Pampori, Volodymyr Gerzanich, J. Marc Simard, Rao P. Gullapalli

**Affiliations:** 1 Departments of Diagnostic Radiology and Nuclear Medicine, University of Maryland School of Medicine, Baltimore, MD, 21201, United States of America; 2 Department of Neurosurgery, University of Maryland School of Medicine, Baltimore, MD, 21201, United States of America; 3 Department of Pathology, University of Maryland School of Medicine, Baltimore, MD, 21201, United States of America; 4 Department of Physiology, University of Maryland School of Medicine, Baltimore, MD, 21201, United States of America; Uniformed Services University, UNITED STATES

## Abstract

Explosive blast-related injuries are one of the hallmark injuries of veterans returning from recent wars, but the effects of a blast overpressure on the brain are poorly understood. In this study, we used *in vivo* diffusion kurtosis imaging (DKI) and proton magnetic resonance spectroscopy (MRS) to investigate tissue microstructure and metabolic changes in a novel, direct cranial blast traumatic brain injury (dc-bTBI) rat model. Imaging was performed on rats before injury and 1, 7, 14 and 28 days after blast exposure (~517 kPa peak overpressure to the dorsum of the head). No brain parenchyma abnormalities were visible on conventional T2-weighted MRI, but microstructural and metabolic changes were observed with DKI and proton MRS, respectively. Increased mean kurtosis, which peaked at 21 days post injury, was observed in the hippocampus and the internal capsule. Concomitant increases in myo-Inositol (Ins) and Taurine (Tau) were also observed in the hippocampus, while early changes at 1 day in the Glutamine (Gln) were observed in the internal capsule, all indicating glial abnormality in these regions. Neurofunctional testing on a separate but similarly treated group of rats showed early disturbances in vestibulomotor functions (days 1–14), which were associated with imaging changes in the internal capsule. Delayed impairments in spatial memory and in rapid learning, as assessed by Morris Water Maze paradigms (days 14–19), were associated with delayed changes in the hippocampus. Significant microglial activation and neurodegeneration were observed at 28 days in the hippocampus. Overall, our findings indicate delayed neurofunctional and pathological abnormalities following dc-bTBI that are silent on conventional T2-weighted imaging, but are detectable using DKI and proton MRS.

## Introduction

Explosive blast-related injuries are one of the hallmark injuries of veterans returning from recent wars. In addition to injuries associated with the primary blast wave, many veterans also suffer from penetrating injuries from explosive fragments and blunt trauma due to mechanical forces to the brain (commonly referred to as blast+TBI, or bTBI) [1]. The effects of penetrating or blunt trauma are relatively well studied, as they share the same injury mechanisms as in the civilian traumatic brain injury (TBI) population [2–4]. However, the effect on the brain of an explosive blast overpressure directly impacting the head, the primary blast injury, is poorly understood.

A significant impediment to understanding the long term effects of the primary blast injury is the lack of a suitable model that incorporates an explosive blast overpressure that directly impacts the head without producing confounding blast related injuries to non-cranial organs. A novel direct cranial blast injury model (dc-bTBI) [5] was recently introduced whose key feature is the delivery of a blast wave to the brain, devoid of the “thoracic mechanism” that also contributes to brain injury [6]. Separating the effects of direct cranial impact of the blast wave from indirect effects on the brain due to blast injury to the remainder of the body is crucial for a comprehensive pathophysiological understanding of bTBI.

Several novel *in vivo* imaging techniques have been explored to study TBI in preclinical models in order to better understand the temporal changes in pathophysiology. Diffusion tensor imaging (DTI) and proton MR spectroscopy (MRS) are both advanced imaging techniques that have shown great promise in identifying subtle biophysical and biochemical changes following TBI. DTI provides information about tissue microstructural changes following TBI [7–9]. Commonly used DTI parameters include mean diffusivity (MD), which measures the average water diffusivity within the tissue, and fractional anisotropy (FA), which provides the preferential direction of water diffusion within a given voxel. A voxel with a value near zero for FA would mean that the probability of a water molecule diffusing in any given direction is about the same, whereas a voxel with a value near unity would mean that the water molecule has a very high preference for diffusing in a particular direction. Early after injury, the MD typically is reduced and the FA is increased, consistent with *reduced* extracellular space due to cellular swelling (cytotoxic edema) [10–12]. This pattern generally reverses during later stages of injury (> 6 months), with the MD increasing and the FA decreasing, consistent with *increased* extra-cellular space due to vasogenic edema, demyelination, cellular membrane disruption and cell death [13–15].

Imaging studies with DTI involving bTBI in both humans [7,16] and experimental animals [17,18] have yielded conflicting results. Levin et al. [16] found no differences in FA or MD values using fiber tracks, region of interest (ROI) or voxel-wise analysis among veterans with mild-to-moderate bTBI, compared to veterans with no exposure to a blast, despite the fact that bTBI patients had residual symptoms and difficulties in verbal memory. By contrast, MacDonald et al. [7] found DTI abnormalities in white matter regions including the middle cerebellar peduncle, the cingulum and orbitofrontal white matter, consistent with traumatic axonal injury in military personnel with a clinical diagnosis of mild uncomplicated bTBI. Even when significant FA changes were reported after bTBI, both increased and reduced FA values have also been reported. Rubovitch et al. [17] reported increased FA in the thalamus and hypothalamus in a mouse model of open field blast that correlated with long term cognitive and recall deficits at 30 days, whereas Budde et al. [18] found reduced FA throughout the cortex, hippocampus and white matter regions in a rat model of primary blast injury around the same time frame (28–31 days) post-blast.

An important limitation of traditional DTI is that it lacks sensitivity in grey matter regions, due to the relatively more isotropic water diffusion profile in grey matter compared to white matter. Furthermore, when shorter diffusion distances are explored through the use of high b-values (diffusion sensitivity factor), the imaging technique becomes increasingly sensitive to heterogeneous cellular microstructures and the assumption that water diffusion has a Gaussian distribution is no longer valid [19,20]. As an extension to the popular DTI model, diffusion kurtosis imaging (DKI) not only provides all diffusion tensor measurements (the Gaussian portion), but also the kurtosis parameters which describes the non-Gaussian water diffusion [19]. Previous studies have shown mean kurtosis (MK), which measures the average kurtosis, to be a marker for tissue complexity or heterogeneity [21–23]. In a recent study in a rat model of controlled cortical impact injury (CCI), our group showed an association between increased reactive astrocytosis and higher MK, despite the absence of abnormalities associated with traditional DTI parameters (both MD and FA) [20].

The molecular cascades that are triggered by the bTBI have not been identified. In contrast, alterations in neurochemistry have been reported to play a key role in impact-related TBI outcomes [12,24–35]. Decreased N-acetylaspartate (NAA) suggestive of neuronal mitochondrial dysfunction or neurodegeneration, elevated lactate (Lac) suggestive of hypoxia, and elevated choline (Cho) and myo-inositol (Ins) implicating membrane breakdown or inflammation [27,33–36] have been reported by several investigators. Altered glutamine (Gln) and glutamate (Glu) also were found in human survivors of TBI [29,30,32]. We reported that metabolic information from proton MRS can complement the microstructural changes observed by DTI in a CCI rat model [12]. Furthermore, in both adult and pediatric populations, changes in certain neurochemicals that are visible with proton MRS have been shown to correlate with injury severity [28], and have been linked with cognitive outcomes in TBI survivors [26,31–33,37].

In this study, we investigated microstructural and metabolic changes in a rat model of dc-bTBI [5].We focused on three regions: the hippocampus, internal capsule and cerebellum, due to their high susceptibility to injury and their high impact on the motor and memory functions in injured animals. We hypothesized that there would be changes in both DKI and MRS signals due to the blast exposure, and that the sequelae of injury would provide unique insights into the effects of a blast wave impacting the brain. We examined the temporal changes in the imaging markers from DKI and MRS by studying dc-bTBI rats at baseline up to 28 days after injury. We also tested the rats for neurofunction at 28 days and examined the relationships between the imaging findings, neurofunction and the resulting histology from this unique injury model.

## Materials and Methods

### dc-bTBI

The experimental protocol was approved by the Institutional Animal Care and Use Committee of the University of Maryland, with IACUC No. 0912012. In all, 30 adult male Long-Evans rats (300 ± 20 gm) were subjected to dc-bTBI using a Cranium-Only Blast Injury Apparatus (COBIA), as described in Keuhn et al. [5]. Briefly, the rats were anesthetized (60 mg/kg ketamine plus 7.5 mg/kg xylazine, IP), intubated with an endotracheal tube, and allowed to breath room air spontaneously. Although focal blast injury per se is not painful, Buprenorphine (0.05 mg/kg) was given s.c. prior to the procedure as preemptive analgesia and every 12 hours for 48 hours after the procedure for relief of potential pain that could arise from the periosteum at the site of the injury. For dc-bTBI, the occipital region of the head was exposed to a blast wave generated by detonating a .22 caliber smokeless powder cartridge, which generated a peak overpressure of~517 kPa (blast dissipation chamber, 24.5 cm; see [5]). Because COBIA is designed to deliver a 25.4 mm collimated blast wave selectively to the occipital region, there is no “exhaust” or “blast wind” injury due to inhalation of hot gases, and there is no transthoracic, transvascular mechanism of injury [6]. All rats subjected to dc-bTBI experienced apnea immediately after blast, but with lethal dc-bTBI, apnea was persistent and was followed within 30–45 seconds by cardiac arrest, while animals were under anesthesia. dc-bTBI resulted in~33% mortality, similar to previous observations [5]. Survivors were nursed on the heating pad until they recovered spontaneous movements. After the recovery from anesthesia (~ 1 hour) rats that underwent dc-bTBI did not exhibit distressful or abnormal behavior different from the behavior of the sham injury animals. Of the 20 surviving post- dc-bTBI rats, 10 underwent serial MR imaging and 10 were used for neurofunctional testing. As controls for neurofunctional testing, an additional 10 rats underwent sham injury, with the entire procedure performed as described above except that the cartridge was not detonated.

Serial MR imaging (see below) was carried out on 10 rats starting from 1 day before the dc-bTBI injury as baseline. Only rats that completed all imaging time points up to 28 days were included for imaging data analysis (n = 6). MR imaging finding of injured rats were compared to a separate group of sham injured rats (n = 6) who went through the same injury preparation without the actual injury, as well as the imaging procedures. Due to concerns regarding the potentially confounding effects of repeated anesthesia used for serial MR imaging, neurofunctional testing (see below) was conducted on a separate group of rats (n = 10) with dc-bTBI that did not undergo serial anesthesia. Neurofunctional performance in dc-bTBI rats was compared to neurofunctional performance in sham injured rats (n = 10). After MR imaging or neurobehavioral testing rats were euthanized with an overdose of sodium pentobarbital (> 100 mg/kg).

### MR Imaging

MR imaging was performed under isoflurane anesthesia before dc-bTBI then at 1, 7, 14 and 28 days after injury. All experiments were performed on a Bruker Biospec 7.0 Tesla 30 cm horizontal bore scanner (Bruker Biospin MRI GmbH, Germany) equipped with a BGA12S gradient system capable of producing pulse gradients of 400 mT/m in each of the three axes, with AVANCE III electronics and interfaced to a Bruker Paravision 5.0 console. A Bruker 4-channel surface coil array was used as the receiver and a Bruker 72 mm linear-volume coil as the transmitter. At all times during the experiment, the animal was under 1–2% isoflurane anesthesia and 1 L/min oxygen administration. An MR compatible small-animal monitoring and gating system (SA Instruments, Inc., New York, USA) was used to monitor the animal’s respiration rate and body temperature. The animal body temperature was maintained at 36–37°C using a warm water bath circulation device. Ear pins were inserted in the rats’ ears to fix their head position in order to reduce head motion. The total duration of the whole imaging experiment was approximately 2 hours.

A three-slice (axial, mid-sagittal, and coronal) scout using rapid acquisition with fast low angle shot (FLASH) was used to localize the rat brain. A fast shimming procedure (Fastmap) was used to improve the B_0_ homogeneity covering the brain. Both proton density (PD) and T_2_-weighted images were obtained using 2D rapid acquisition with fast spin echo sequence in both the axial and coronal planes. Imaging was performed over a 3 cm field of view (FOV) in the coronal plane with an in-plane resolution of 117μm using 24 slices at 1 mm thickness, at an effective echo-time of 18.9 ms for the proton density weighted images and an effective echo-time of 56.8 ms for the T_2_-weighted images. The echo-train length for the fast spin echo sequence was 4, the repeat time (TR) was 5500 ms, and the total acquisition time was ~12 minutes using two averages.

For the DKI acquisition, diffusion weighted images were acquired with a single shot, spin-echo echo-planar imaging (EPI) sequence. An encoding scheme of 30 gradient directions was used with the duration of each of the diffusion gradients (δ) being 4 ms with a temporal spacing of 23 ms (Δ) between the two diffusion gradients. Three b-values (1000 s/mm^2^, 1500 s/mm^2^ and 2000 s/mm^2^) were acquired for each direction following the acquisition of 5 images acquired with b = 0 s/mm^2^. The DKI images were obtained using 2 averages over the same FOV as the axial PD/T_2_ images but at an in-plane resolution of 0.234 mm at a TR/TE of 6000/50ms respectively for a total acquisition time of ~21 min.

**Fig 1:**
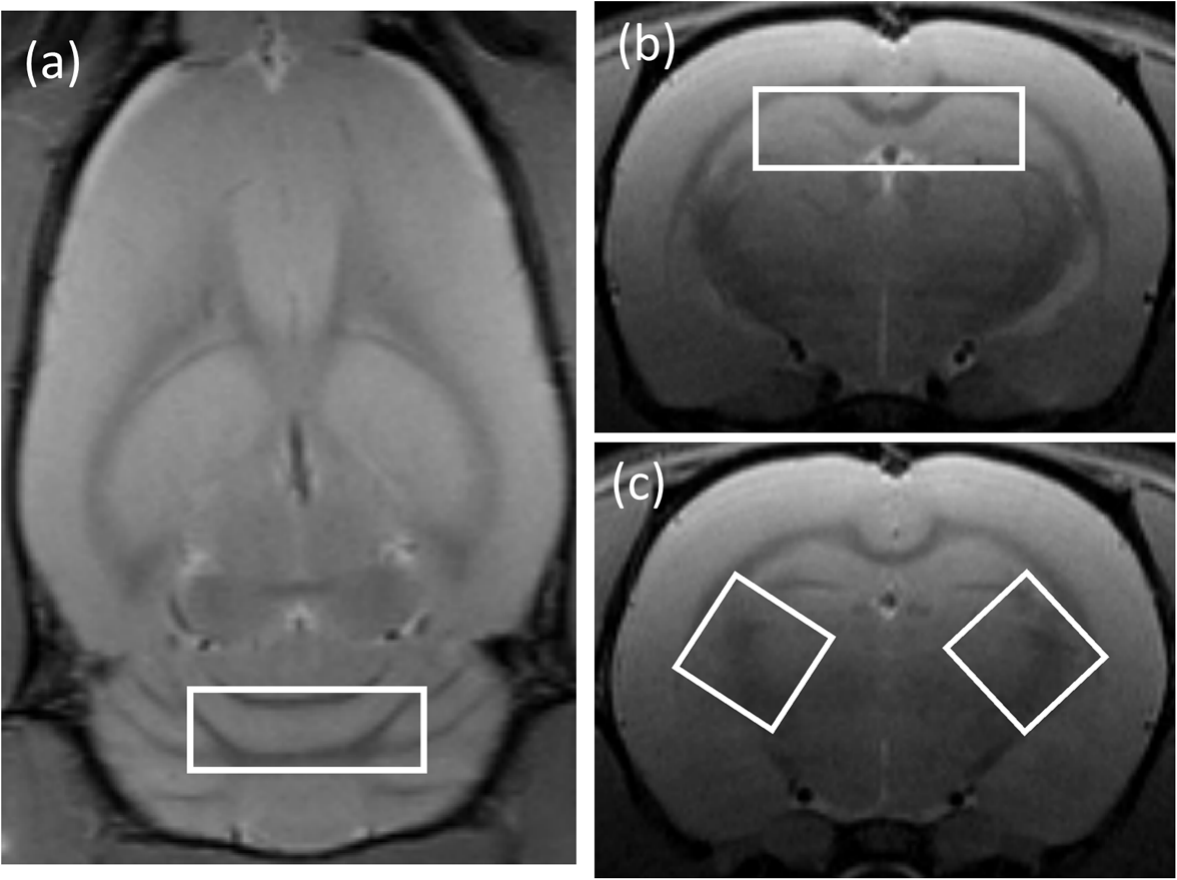
Placement of the MRS voxels (white contours) on (a) cerebellum, (b) hippocampus, and (c) bi-lateral internal capsule on representative MR images of a rat.

For ^1^H MRS, adjustments of all first- and second-order shims over the voxel of interest were accomplished with the FASTMAP procedure. The *in vivo* shimming procedure resulted in full width half maximum (FWHW) ranging from 7.8–10.9 Hz for the unsuppressed water peak in the spectroscopy voxel of the rat brain. This value is comparable with a Bruker spherical quality assurance phantom which typically results in a 5 Hz FWHM. The water signal was suppressed by variable power radiofrequency (RF) pulses with optimized relaxation delays (VAPOR). Outer volume suppression (OVS) combined with point-resolved spectroscopy (PRESS) sequence from a 3 x 3 x 3 mm^3^ voxel was used for signal acquisition, with TR/TE = 2500/20 ms, spectral bandwidth = 4 kHz, number of data points = 2048, number of averages = 300. Spectral data were obtained from two voxels that covered bi-lateral internal capsule, and two other voxels that covered the center of the hippocampus and the cerebellum respectively (Fig 1).

### Image Analysis

The DKI data were first smoothed using 3D Gaussian smoothing with FWHM = 0.3 mm to improve the signal-to-noise ratio (SNR). DKI reconstruction was performed on each voxel using in-house MATLAB program (Mathworks, Natick, MA), as described by Zhuo et al. [20]. The standard most-widely used DTI model uses the following equation (Eq 1) to arrive at the tensor:

$$\text{DTI}:\;ln\left[S\left(b\right)/S_{0}\right]=-bD_{app}$$ (1)

Where *D*_*app*_ is the apparent diffusion coefficient (mm^2^/s)in a given diffusion direction, *S(b)* is the diffusion weighted signal along that direction, b is the diffusion sensitivity parameter (s/mm^2^), and *S*_*o*_ is the MR signal intensity when no diffusion-weighting is applied. On the other hand, the DKI model uses the following equation [19]:

$$\text{DKI}:\;ln\left[S\left(b\right)/S_{0}\right]=-bD_{app}+\frac{1}{6}b^{2}D_{app}^{2}K_{app}$$ (2)

where *K*_*app*_ is the kurtosis along a certain diffusion direction. In probability theory, kurtosis is a term to describe the “peakness” of a probability distribution, as compared to a Gaussian “bell shaped” distribution. In the DKI model (Eq 2) the extra b^2^ term and *K*_*app*_ captures the deviation of the water diffusion from a mono-exponential form as predicted by the theoretical Gaussian model (as in DTI), especially when higher b-values are used.

**Fig 2:**
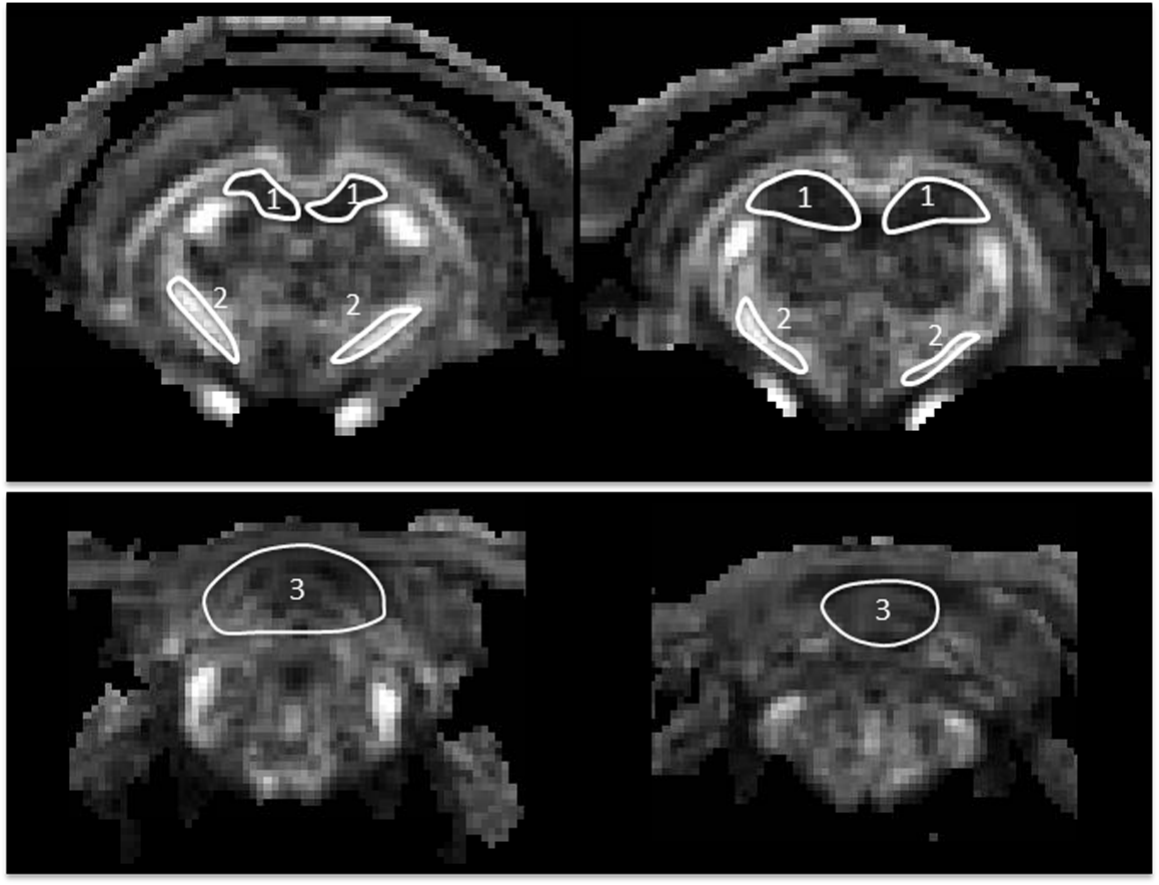
Placement of DKI ROIs (white contours) on coronal FA maps for bi-lateral hippocampus (1), internal capsule (2), and cerebellum (3).

Maps of MD, FA, and mean kurtosis (MK) were generated using Eq 2. MK was calculated as the average of *K*_*app*_ along all measured diffusion directions. Manually drawn regions of interest (ROI) were placed bi-laterally in the internal capsule, hippocampus and cerebellum, corresponding to the regions where MRS data were obtained. The DKI ROIs were drawn on 2–3 consecutive slices on FA images in combination with MD images to ensure no CSF area was included. In addition, T2w images were used as anatomical references guided by the rat brain atlas (Pixnos and Watson, 1986) (Fig 2). Mean MD, FA, MK values from each of the ROIs were measured. Post-injury MRI data were normalized by the corresponding baseline data for each rat.

^1^H MRS data were fitted using the LC Model package [38]. Cramér-Rao lower bounds (CRLB), as reported from the LCModel analysis, were used to assess the reliability of measures of transmitters and the major metabolites. Metabolites having CRLB values less than 20% were further analyzed. The *in vivo* mean metabolite concentrations relative to total creatine (tCr) at each time point were computed.

Since the blast wave was directed to impact mid-sagittally with no lateral preference, the MRS and DKI data from bilateral regions were averaged to improve signal sensitivity.

### Neurofunctional Tests

Vestibulomotor functions were assessed on days 1–14 following dc-bTBI using the beam walk test, the beam balance test, and the accelerating RotaRod test [39–41]. In the beam walk test, the rat was timed as it walked a 90 × 2 cm beam to a darkened box, while a loud noise was created behind it. In the beam balance test, the ability of the rat to balance on a 30 × 1.5 cm beam was scored for each trial, based on criteria described by Petullo et al. [39] Baseline values were collected after 2 days of training, which consisted of 3 trials per day. In the accelerating RotaRod test, rats were placed on the drum of the accelerating RotaRod (IITC, Life Science, Woodland Hills, CA), starting at 4 rpm, accelerating at a rate of 2 rpm every 5 sec up to a maximum of 45 rpm; 3 trials separated by 20 min were administered on each day of testing, with the average latency to falling off of the drum being reported.

Exploratory behavior was measured by assessing spontaneous rearing using the cylinder test [42–45]. Rats were placed in a plexiglass cylinder (10 cm diameter, 20 cm height) for 3 min. The time spent rearing and exploring the walls with their vibrissae and forelimbs was recorded.

Incremental and rapid spatial learning were measured using the Morris Water Maze (MWM) after testing vestibulomotor function to ensure that deficits observed in dc-bTBI animals early after injury minimally interfere with behavior in MWM as significant motor deficits prevent objective assessment of the performance in MWM. The MWM device and the specific paradigms used in this laboratory have been described in detail [46,47]. Learning the location of the hidden platform from distant visual cues during 5 successive days of training, which is referred to as “incremental spatial learning”, took place on days 14–18 after dc-bTBI or sham-injury. On the following day, day 19 after dc-bTBI or sham-injury, rats were tested for memory retention during a probe trial because probe trial performance has been shown to be a more accurate measure of cognitive function than the incremental learning trials [48]. Then, on day 21, rapid learning was tested by moving the hidden platform to a new quadrant and giving the rats a single learning trial before the probe trial. The time spent in the correct (new) quadrant, which should be more than 25% based on chance alone, is taken as a measure of their ability to rapidly learn the new position. The ability for one-trial rapid learning is a hippocampus-specific cognitive function requiring place information to traverse the full trisynaptic pathway (entorhinal cortex → DG → CA3 →CA1 → entorhinal cortex) [49]. In addition, the feed-forward pathway from the entorhinal cortex to the DG and on to CA3 is needed for pattern separation [50].

### Histochemistry

For the dc-bTBI and sham-injured animals that underwent neurofunctional testing, rats were euthanized on day 28 using a lethal dose of pentobarbital IP(> 100mg/kg), and the brains were perfusion-fixed with 10% neutral buffered formalin. After fixation, the brains were cryoprotected using 30% sucrose in PBS for 48 hr at 4°C.

For immunolabelling, coronal cryosections (10 μm), obtained at –5.5 to –6 mm from bregma, were blocked with 5% goat serum (G-9023; Sigma Aldrich, St. Louis, MO) plus 0.2% Triton X-100 (T8787; Sigma-Aldrich) for 1 hour at room temperature. Sections were then incubated overnight at 4°C with rabbit anti-ionized calcium binding adaptor molecule 1 (Iba1) (1:1000; 019–19741; Wako Chemicals, Richmond, VA). After several rinses in phosphate buffered saline, the slides were incubated for one hour with fluorescent-labeled anti-rabbit secondary antibody (1:500; A-11034; Alexa Flour 488; Invitrogen, Molecular Probes, Eugene, OR) at room temperature. Omission of primary antibody was used as a negative control. The sections were cover-slipped with polar mounting medium containing antifade reagent, ProLong Gold AntifadeMoutant with 4’,6-diamidino-2-phenylindole (DAPI; P36931; Invitrogen, Eugene, OR), and were examined using epifluorescence microscopy (Nikon Eclipse 90i; Nikon Instruments Inc., Melville, NY).

To identify degenerating neurons, cryosections (10 μm) were labeled with Fluoro-Jade C histo-fluorescent stain [51] according to the manufacturer’s protocol (1FJC; Histo-Chem, Jefferson, AR). Sections were cover-slipped with non-polar mounting medium (8312–4; Cytoseal XYL, Richard-Allan Scientific, Kalamazoo, MI), and were examined using epifluorescence microscopywith a fluorescein isothiocyanate (FITC) filter, with care being taken to limit the time of exposure in order to reduce photo bleaching.

Unbiased measurements of specific labeling within an ROI were obtained using NIS-Elements AR software (Nikon Instruments, Melville, NY) from sections immune-labeled or stained in a single batch, as previously described [46,52]. All ROI images for a given signal were captured using uniform parameters of magnification, area, exposure, and gain, and were applied to multiple sections. The following ROIs were defined: (i) for Iba1 in the hippocampus, a rectangular ROI, 450 ☓ 360 μm, was positioned on the perforant pathway; (ii) for Fluoro-Jade C in the hippocampus, a rectangular ROI, 450 ☓ 360 μm, was positioned at the hilus of the dentate gyrus. Segmentation analysis was performed by computing a histogram of pixel intensity for a particular ROI. Specific labeling was defined as pixels with signal intensity greater than 2☓ that of background (standard threshold based on the analysis of the intensity histograms of the ROI’s with no labeling). For both Iba1 and Fluoro-Jade C, the area occupied by pixels with specific labeling was used to determine the percent area with specific labeling (% ROI).

### Statistical Analysis

Statistical calculations were carried out using either SPSS (IBM SPSS, version 21) or OriginPro8 (OriginLab Corp., Northampton, MA, USA). MRI data from the two groups of TBI and the sham at 5 time points and neurofunctional data were analyzed using a two-way repeated measure ANOVA with Fisher’s LSD post-hoc comparisons (6 rats/group for MRI, 10 rats/group for neurofunctional, for a balanced design). Non-parametric datasets (beam walk scores) were rank-transformed prior to the application of parametric statistical analysis. Histochemical data were analyzed using an unpaired t-test. Statistical significance was accepted if *p*<0.05.

## Results

### MR Imaging

**Fig 3:**
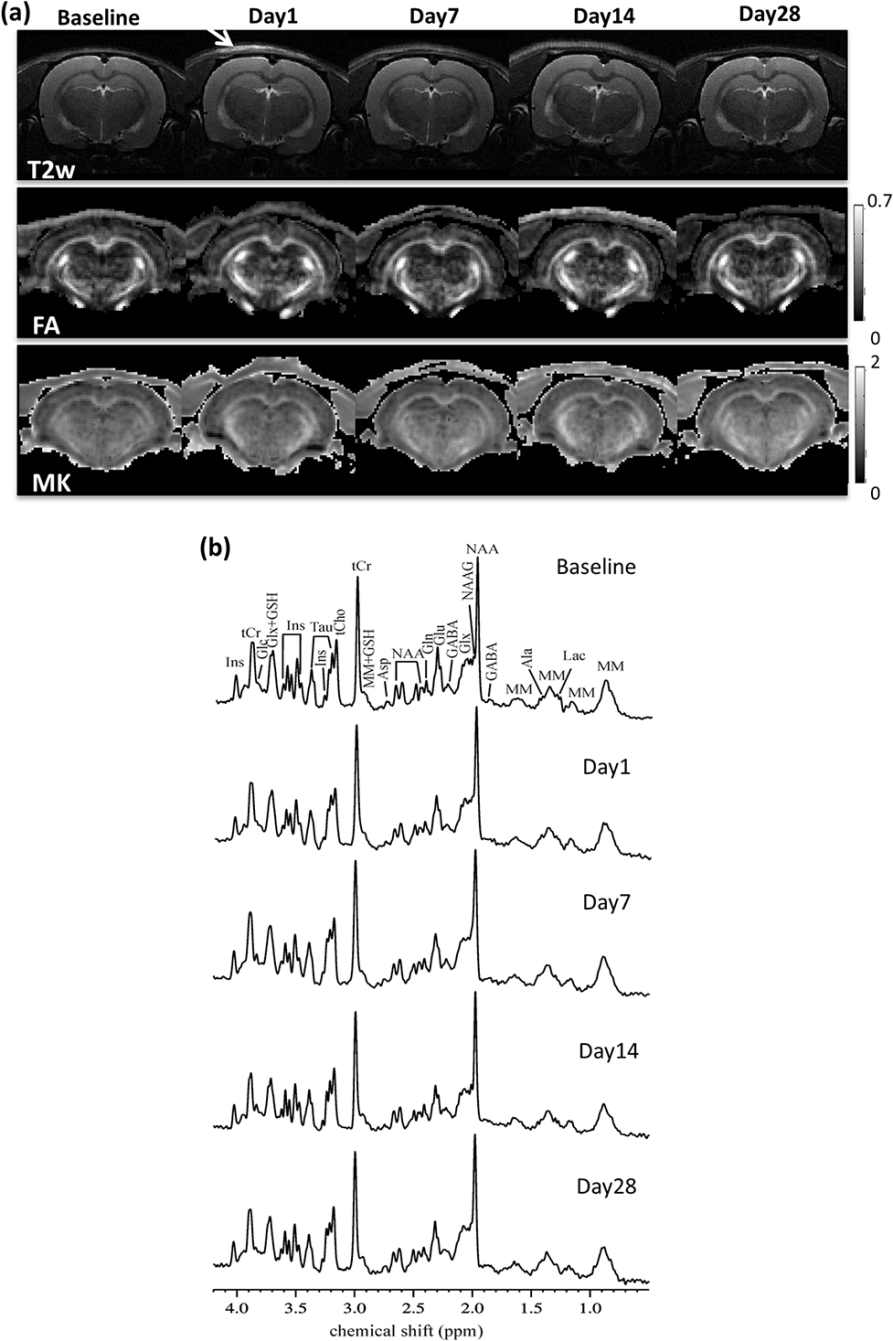
MRI data of a representative dc-bTBI rat at different time points from baseline to 28 days post injury. (a) T2w images, FA and MK maps from DKI for a coronal slice. Arrow indicated soft tissue contusion from the injury, which were apparent at Day1 on T2w images but has fully resolved by Day7. (b) In vivo proton MRS acquired in the hippocampus. alanine (Ala), asparttate (Asp), g-aminobutyric acid (GABA), glucose (Glc), glutamine (Gln), glutamate (Glu), glutathione (GSH), myo-inositol (Ins), lactate (Lac), N-acetylaspartate (NAA), N-acetylaspartylglutamate (NAAG), taurine (Tau), total creatine (tCr), choline compounds (tCho), glutamate/glutamine complex (Glx), and macromolecules (MM).

Fig 3 shows representative *in vivo* T2w, DKI, FA and MK images (Fig 3A) and MRS spectrum (Fig 3B) for a representative dc-bTBI rat at all imaging points from baseline to 28 days post injury. The location of the dc-bTBI injury was visible at Day1 on T2w images, which appeared to be fully resolved by Day7. No other abnormalities within the brain parenchyma were observed at any imaging time point.

**Fig 4:**
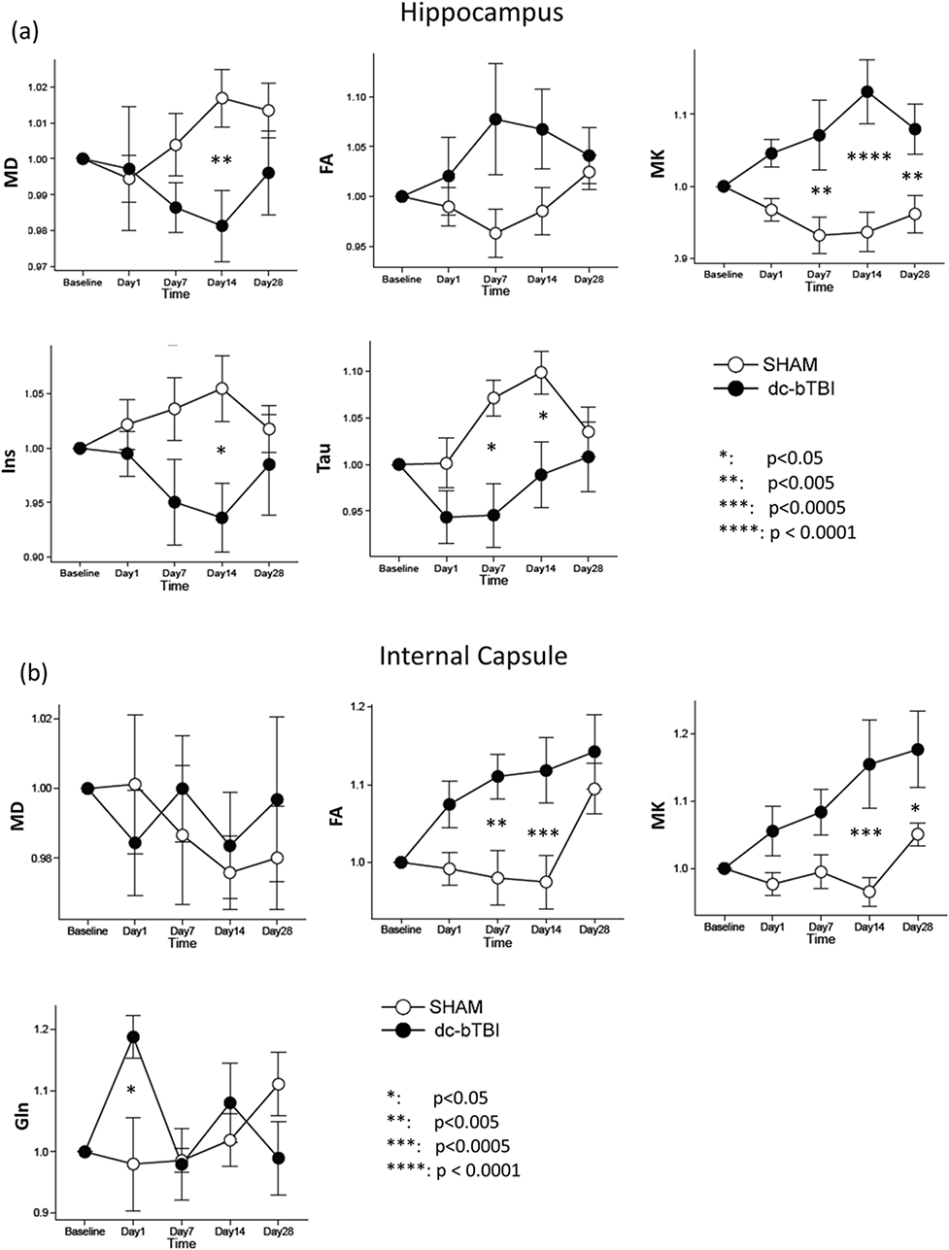
Changes of DKI and MRS parameters in the sham and dc-bTBI rats in the hippocampus (a) and internal capsule (b) from baseline to 28 days post injury. Temporal data in each rat were normalized to its baseline (pre-injury) value. Error bar shows standard error. Comparisons were made between sham and dc-bTBI rats.

In the hippocampus (Fig 4A), no abnormalities were observed in T2w images. However, DKI results indicated significant group effect between dc-bTBI and sham rats for MK (F = 11.384, p = 0.007). Significant group-by-time interaction was found for MK (F = 6.424, p = 0.0004) and MD (F = 3.07, p = 0.027). Post-hoc tests indicated that dc-bTBI rats had significantly higher MK at 7 days (p = 0.0006), 14 days (p < 0.0001) and 28 days (p = 0.004), with a clear trend of maximum difference at 14 days and recovery at 28 days. Reduced MD was accompanied by MK increase, although MD was only significant at 14 days (p = 0.005). MRS results showed significant group difference between dc-bTBI and sham rats in Tau (F = 6.7, p = 0.027) and Ins (F = 5.4, p = 0.042) levels. Significant group-by-time interaction was found in Tau (F = 3.0, p = 0.029). Post-hoc tests indicated that dc-bTBI rats had significantly lower Tau level at 7 days (p = 0.004) and 14 days (p = 0.011), as well as lower Ins level at 14 days (p = 0.020).

Similar DKI changes were observed in the internal capsule (Fig 4B), although in this typical white matter region, FA showed significant changes concomitant with MK changes, while there were no changes in MD. In this region, MK had both a significant group effect (F = 6.37, p = 0.030), and a significant group-by-time interaction (F = 4.37, p = 0.005). Significant group-by-time interaction was observed for FA (F = 4.62, p = 0.004). Post-hoc tests indicated that dc-bTBI rats had significantly higher MK at 14 days (p < 0.001) and 28 days (p = 0.026); as well as significantly higher FA at 7 days (p = 0.0032) and 14 days (p < 0.001), with both demonstrating a trend for peak increase at 14 days and recovery at 28 days. MRS results showed significant group-by-time interaction in the Gln level (F = 3.89, p = 0.009), where the dc-bTBI rats showed significantly higher Gln level at 1 day post injury (p = 0.016).

There were no significant group effects or group-by-time interactions found in any MRI or MRS measures of the cerebellum. All imaging data are provided in S1 File.

### Neurofunction

**Fig 5:**
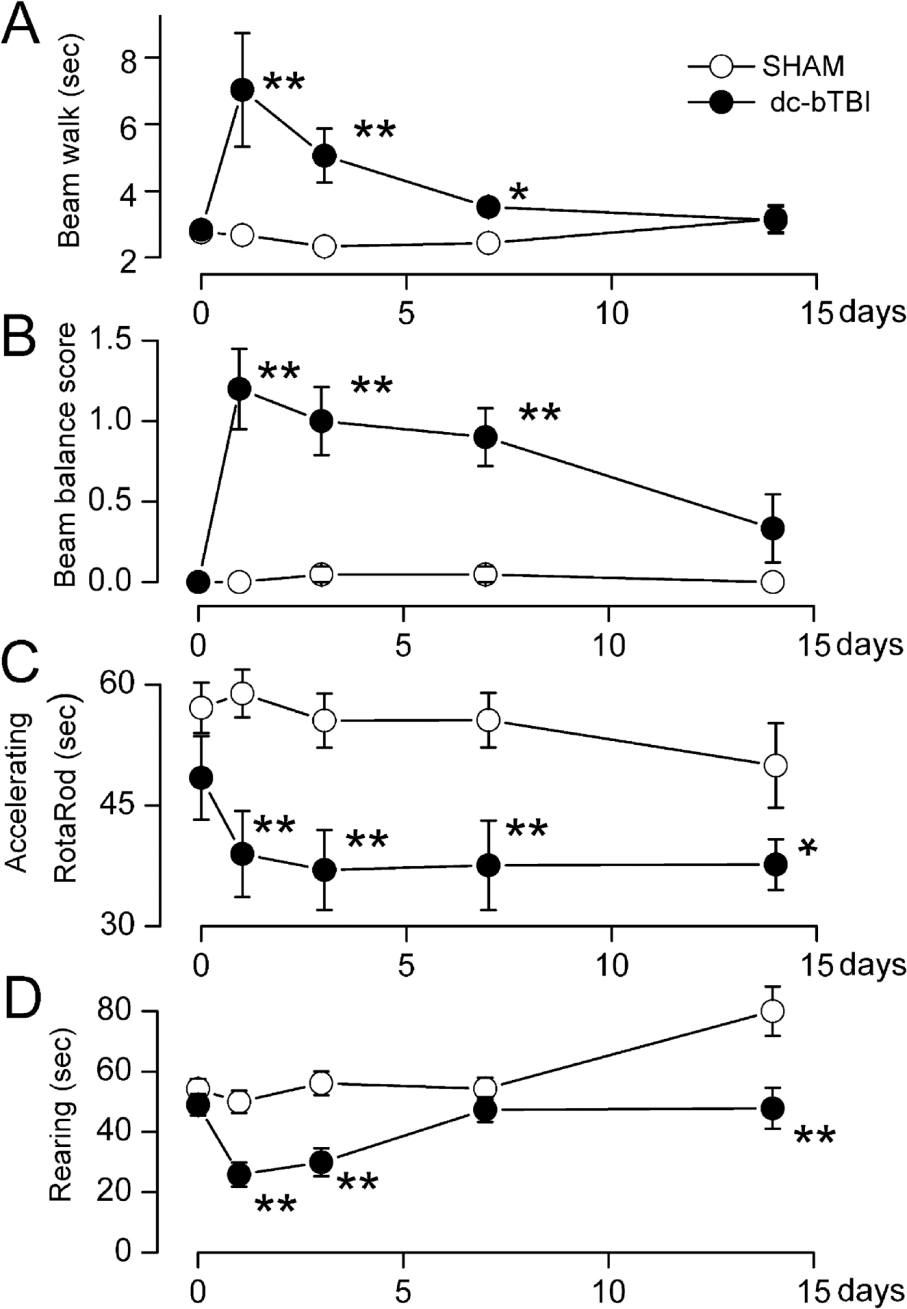
dc-bTBI causes abnormalities in vestibulomotor performance. A–D: performance on beam walk (A), beam balance (B), accelerating RotaRod (C) and spontaneous rearing (D) over the course of 1–14 days; n = 10 sham-injury, 10dc-bTBI; day 0 is baseline before dc-bTBI;*, *p*< 0.05; **, *p*<0.01for comparison between sham and dc-bTBI rats.

The rat model of dc-bTBI that we studied was associated with abnormalities in vestibulomotor function, complex neuromotor function, and neurocognition, with vestibulomoter abnormalities being prominent early after injury. Compared to the sham-injured group, dc-bTBI rats performed significantly worse on the beam walk test at 1 and 3 days (p< 0.01), with partial recovery at 7 days (p = 0.014) after injury (Fig 5A). Compared to sham-injured animals, dc-bTBI rats performed significantly worse on the beam balance test at 1, 3 and 7 days (p< 0.01) (Fig 5B), and fell off the accelerating RotaRod sooner at 1, 3 and 7 days (p< 0.05) (Fig 5C). The rearing time was significantly shorter in dc-bTBI rats at 1 and 3 days (p< 0.01), and partially recovered at 7 days after dc-bTBI (Fig 5D).

**Fig 6:**
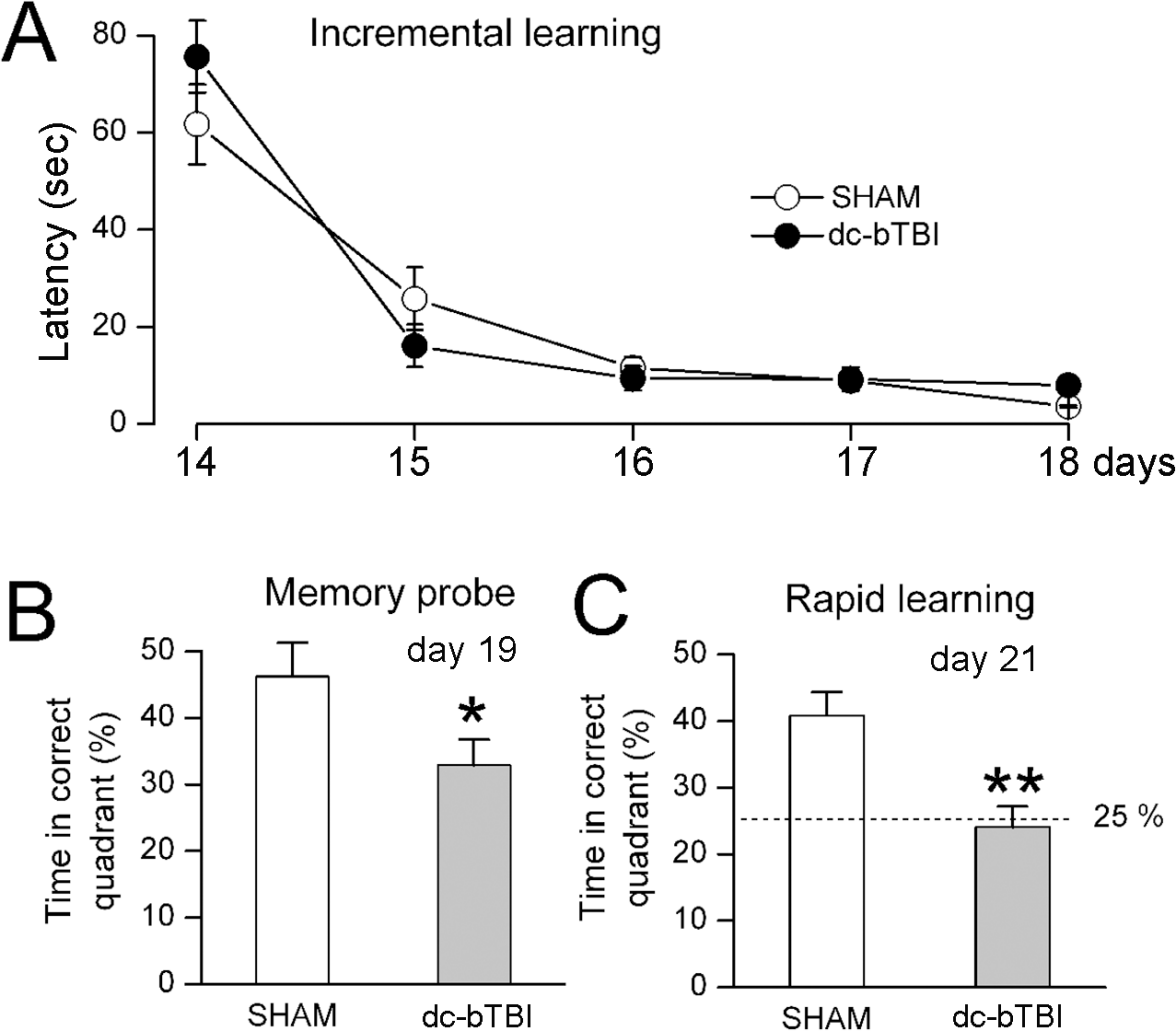
dc-bTBI causes abnormalities incognitive function measured using Morris Water Maze test. Performance during incremental learning (A), memory probe test (B), and rapid learning test (C) during days 14–21, as indicated; n = 10 sham-injury, 10 dc-bTBI; in (C), 25% represents chance alone;*, p< 0.05; **, p<0.01 for comparison between sham and dc-bTBI rats.

Cognitive abnormalities were prominent later after injury (Fig 6). No differences were identified in incremental learning during days 14–18 after dc-bTBI (Fig 6A). However, significant deficits were found on the memory probe on day 19 and on the rapid learning test on day 21 after dc-bTBI (Fig 6B and 6C).

### Histology

**Fig 7:**
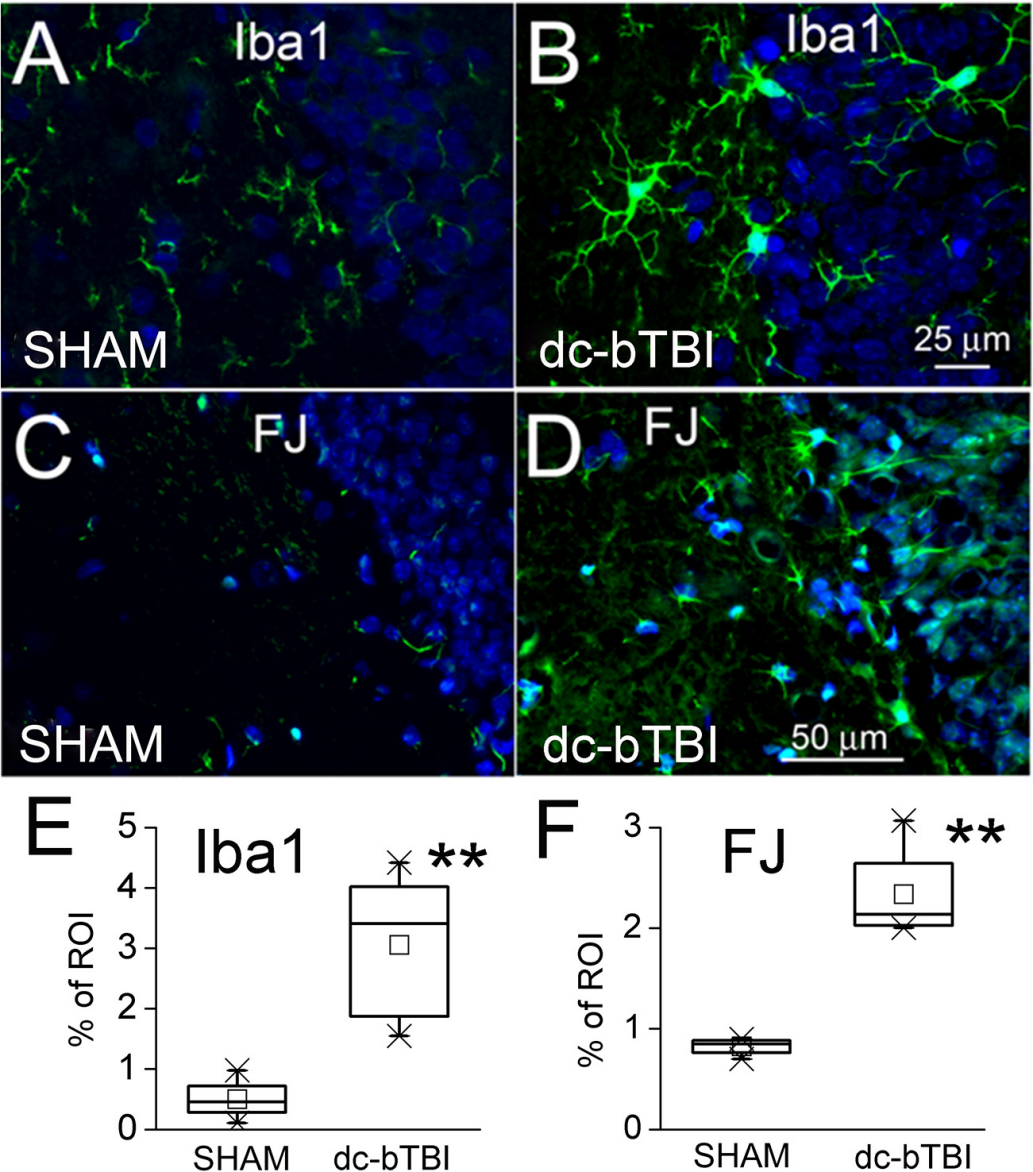
Histochemical findings in the hippocampus in dc-bTBIvs. sham-injured rats at 28 days.A,B: Iba1 immunolabeling (green) for microglia in a sham-injured and a dc-bTBI rat,respectively. C, D: Fluoro-Jade C (green) for neurodegeneration in a sham-injured and a dc-bTBI rat, respectively. E,F: Quantification of Iba1 immunolabeling and Fluoro-Jade C staining in sham-injured and dc-bTBI rats; in A–D, DAPI (blue) nuclear labeling is also shown; n = 5 per group; **, *p*<0.01for comparison between sham and dc-bTBI rats.

On day 28 after dc-bTBI, the brains of animals that had undergone neurofunctional testing were examined for evidence of hippocampal injury. We focused on the hippocampus because deficits in rapid spatial learning and memory are considered to be hippocampus-specific [49,50,53,54]. Immunolabeling for Iba1 showed significant microglial activation in the hippocampus (Fig 7). Also, staining with Fluoro-Jade C showed significant neurodegeneration in the hippocampus (Fig 7).

## Discussion

Primary blast injury is often associated with other blast related injuries, including penetrating injury, tertiary injury from high velocity air flow that results in acceleration/deceleration injuries, and quaternary injuries with hemorrhagic or chemical shock [55]. Of these and other blast related injuries, the primary blast injury is the least well understood. In this study we assessed, for the first time *in vivo*, pathophysiological changes in a well characterized dc-bTBI model [5]. The findings of this study indicate that: (i) although no apparent injuries were detected with conventional T2-weighted imaging, both microstructural (DKI) and metabolic (proton MRS) changes could be identified in the hippocampus and internal capsule, but not in the cerebellum, following dc-bTBI; (ii) profound DKI and MRS alterations were observed in the hippocampus that peaked at 14 days with sustained MK abnormalities still present at 28 days. These *in vivo* results were in line with neurofunctional and pathological abnormalities associated with hippocampal injury; (iii) DKI changes in the internal capsule were accompanied by transient changes in MRS as early as 1 day post injury, and were associated with recovery of the vestibulomotor function; and (iv) both DKI and MRS changes are suggestive of abnormal astrocyte/glial activity or inflammation in the affected regions. Overall, our findings suggest that relatively subtle brain abnormalities induced by dc-bTBI can be detected by DKI and proton MRS.

Sub-lethal blast injury created in our model of dc-bTBI resulted in reduced MD in the hippocampus and increased FA in the internal capsule. Damage to both the internal capsule and hippocampus has been identified frequently in diffuse axonal injury for both blast and non-blast related TBI. The increase in FA typically is attributed to cytotoxic edema, axonal swelling, or a disruption of ionic homeostasis resulting in an imbalance in intra- and extra-cellular water [9,11]. In previous studies on a rat model of CCI [12,20], we also observed a MD reduction in the hippocampus and FA increase in white matter regions at 2 hours after injury, which normalized as early as 4 hours in the contra-lateral regions, and by 7 days in the ipsi-lateral cortex. In contrast to the more transient FA/MD changes in the CCI related TBI, a delayed change in the FA and MD at 14 days was observed in the dc-bTBI model. These delayed DTI responses have been reported in a few other bTBI animal models which suggest that changes may depend on injury severity. In a mouse model of mild open field blast injury, Rubovitch et al. [17] found increased FA in the thalamus and hypothalamus, which peaked at 7 days for the low exposure group, while lasting to 30 days in the high exposure group. They also observed correlative disruption of the blood-brain barrier and changes in expression of oxidative stress markers. In another study of the primary blast TBI effect in a rat model using a shock tube and restrained head motion, Budde et al. [18] also found increased FA in white matter regions at 4 days post injury, which appeared to normalize by the 30 day time-point. In gray matter regions of cortex and hippocampus they found mostly reduced FA voxels starting ipsilateral to the blast wave at 4 days post injury and extending to the contralateral hippocampus by 30 days after injury. The total number of abnormal FA voxels (increase or decrease) correlated with the significant memory deficits in the bTBI rats.

In addition to changes in FA and MD, we also observed an accompanying increase in the MK in both the internal capsule and hippocampus. Changes in MK were robust and, while peaking at 14 days, appeared to last for 28 days post injury. Increased MK is an indication of increased water diffusion heterogeneity [19], which suggests a more complex and varied microenvironment. In our previous study using DKI in the rat CCI model, we observed an increase in MK in the contralateral cortex at 7 days after injury, despite the fact that other parameters such as FA and MD had normalized by 7 days after demonstrating transient changes. The increased MK at 7 days was associated with increased reactive gliosis observed on histopathology [20]. Indeed, gliosis has been identified as prominent pathology after blast injury [56] and could last well into 30 days post injury [18]. In the present study, we observed increased microglial activation in the hippocampus, which was accompanied by cellular damage, as indicated by the Fluoro-Jade C staining 28 days after dc-bTBI. In a recent study conducted on a rat model of CCI, using Fourier analysis of histology images, it was shown that the contribution of gliosis to the observed increased diffusion anisotropy was significant, due to the coherent organization of reactive astrocytes [18]. It is conceivable that the observed FA increase in the internal capsule in the present study may have been driven by reactive gliosis, but additional studies would be required to determine the exact biological process involved.

The indication of reactive gliosis from DKI is also supported by the MRS data. Tau and Ins are well known glial markers. Astrocytes are virtually the sole site for Gln syntheses activity in the brain to convert glutamate to Gln [57,58]. Gln synthesis in astrocytes effectively protects neurons against blood-derived ammonia and glutamine is the end-product of ammonia detoxification [59]. Zwingmann et al. [60] showed that ammonia exposition on glioma cells could elevate Gln and reduce Tau and Ins. Glial cells experience a series of complicated temporal changes in cell volume, including rapid cell swelling followed by regulatory cell volume decrease. The DKI alterations found in our current study may reflect this complex water homeostasis change in both the hippocampus and the internal capsule. Therefore, it is reasonable to hypothesize that dc-bTBI may cause a local hyperammonemia condition resulting in the observed changes in water homeostasis.

Of special note is that the MRS abnormality of Tau and Ins was observed to peak at 14 days in the hippocampus, while only glutamine alterations were observed in the internal capsule transiently at 1 day post injury. This response may be reflective of the differential response depending on the level of injury experienced, with more severe injury in the hippocampus and a milder response to the injury in the internal capsule. Indeed, faster recovery was observed on the simple and complex locomotor functions by 15 days in the dc-bTBI rats which may explain the transient findings in the internal capsule. The internal capsule contains the corticospinal tracts that carry motor information from the primary motor cortex to the lower motor neurons in the spinal cord, and lesions in the internal capsule are often associated with motor function deficits [61]. Taken together, these findings likely indicate that the dc-bTBI may have resulted in a less severe white matter injury, as also found by Budde et al. [18] with a similar rat model for primary blast effect. Although the dc-bTBI rats did not differ from the sham animals on the incremental learning task through the early period post injury, they performed poorly in Morris Water Maze tests that probed their collection of incrementally-learned as well as rapidly-learned memory, assessed 19–21 days after blast exposure. The hippocampus is known to relate to memory functions [50,53,54], and alterations of FA signals in the hippocampus previously were linked to functional memory impairments in bTBI rats [18]. Prolonged hippocampal abnormality was confirmed with both the MK abnormality, as well as histological alterations as late as 28 days post injury. Whether the memory function recovers over longer periods of observation remains to be discovered. Overall, while the neurofunctional and histological changes were similar as observed by our previous study using the same injury model [5] and were associated with imaging studies, future studies focused on longer periods of observation beyond 28 days might provide more insights into the sequelae from dc-bTBI.

While the findings of this study clearly indicate neurofunctional and pathological abnormalities following dc-bTBI that are silent on conventional T2-weighted imaging, but detectable using DKI and proton MRS, they should be viewed in the context of the limitations of this study. First, the neurofunctional and neuroimaging studies were performed on separate sets of animals, which limited our ability to perform direct correlations at each time point. However, it should be noted that the neurofunctional data are consistent with the existing literature, including a report from our group using the same injury model [5]. A second limitation is the lack of quantitative histology, which would have allowed for a direct comparison with *in vivo* imaging findings. Future studies would benefit from direct correlation with quantitative histology, which will be very beneficial in the appropriate interpretation of the advanced *in vivo* imaging data.

## Conclusion

Our data from DKI and MRS analysis indicate that both microstructural and metabolic changes occur following dc-bTBI. These changes develop gradually, resulting in pronounced effects 14–28 days after dc-bTBI, and are consistent with other studies reporting delayed structural changes in bTBI. Increased MK, together with reduced Ins, Tau, Cho and Gln levels, may be indicative of astrocyte abnormality after bTBI. Our results demonstrate that the sequalae of dc-bTBI may be appreciably different from traditional blunt impact TBI. While advanced imaging techniques provide valuable information on the metabolic and microstructural changes, further studies will be needed to relate such changes to alterations in function and neurovascular coupling,and to obtain a better appreciation of the pathophysiology following bTBI.

## Supporting Information

S1 File MRI imaging data for all animals. (XLS)
